# Proteinuria: From Molecular to Clinical Applications in Glomerulonephritis

**DOI:** 10.1155/2012/424968

**Published:** 2012-08-22

**Authors:** Claudio Bazzi, Omran Bakoush, Loreto Gesualdo

**Affiliations:** ^1^D'Amico Foundation for Renal Diseases Research, 20145 Milan, Italy; ^2^Department of Nephrology, Lund University, 22/85 Lund, Sweden; ^3^Department of Internal Medicine, UAE University, Al Ain, UAE; ^4^Section of Nephrology, Department of Emergency and Organ Transplantation, University of Bari, 70124 Bari, Italy

Glomerular diseases including diabetic nephropathy are the leading cause of ESRD worldwide. Proteinuria, the hallmark of renal damage in glomerular diseases, is dependent on two main factors: the alteration of the glomerular filtration barrier and its three layers (glomerular endothelial cells, basement membrane, and visceral epithelial cells (podocytes)), and the impairment of proteins reabsorption by proximal tubular epithelial cells. In recent years there has been a great increase of knowledge of the molecular structure of glomerular filtration barrier and of the molecular mechanisms involved in tubular reabsorption of proteins.

In this special issue the structure of the glomerular filter and the importance of glomerular talk in maintaining the integrity of the glomerular filtration barrier are reviewed by M. Menon et al. A. Tojo and S. Kinugasa reviewed the albumin glomerular permeability in normal and disease status and present their views of a possible mechanism of selective proteinuria in nephrotic syndrome of minimal change disease. A. Zhang and S. Huang summarized several molecular defects responsible for dysfunction of the glomerular filtration barrier. J. E. Toblli et al. overviewed the alterations of glomerular endothelial cells, basement membrane and podocytes, the possible relationship between glomerular proteinuria and tubulointerstitial damage, and described less and more recent approaches to reduce proteinuria. The review of J. R. Machado et al. summarizes the most important molecules involved in the pathogenesis of nephrotic syndrome. Galactose-deficient IgA1 is the hallmark of IgA nephropathy; in a cohort of 40 pediatric patients with biopsy-proven IgAN, a research group from the Le Bonheur Children's Hospital found no association between albuminuria and the galactose-deficiency, a finding that may question the pathologic role of galactose-deficiency in IgAN. The possible therapeutic efficacy of inhibition of mammalian target of rapamycin (mTOR) in primary mesangioproliferative glomerulonephritis was discussed by H. Trimarchi et al. who suggested prospective clinical trials. B. Zhang and W. Shi reviewed the therapeutic effects of cyclosporine A (CsA) in glomerulonephritis and evaluated the data in support of a nonimmunologic antiproteinuric effect of CsA dependent on a direct stabilization of podocyte cytoskeleton. The review by A. Cohen-Bucay and G. Viswanathan discusses nine additional urine biomarkers that may offer better prediction for the course of diabetic kidney disease progression than urinary albumin. The authors call for further longitudinal studies to validate the clinical value of these biomarkers to overcome the limitations of albuminuria.

The great increase in knowledge of molecular biology of glomerular filtration barrier and tubular reabsorption of proteins has not been matched as yet by outcome prediction improvement and therapeutic advances. There is a need for further studies for better understanding the pathogenesis of proteinuria and the clinical value of the different urine biomarkers in diagnosis and management of patients with chronic glomerular diseases.

Finally special thanks to the authors and the reviewers for their efforts to provide to the nephrology community a concise up-to-date knowledge on the pathogenesis and management of proteinuric kidney disease.

*Claudio Bazzi* *Omran Bakoush* *Loreto Gesualdo*

